# Green synthesis and characterization of silver nanoparticles through the *Piper cubeba* ethanolic extract and their enzyme inhibitory activities

**DOI:** 10.3389/fchem.2023.1065986

**Published:** 2023-02-22

**Authors:** Khalil Ahmad, Hafiz Muhammad Asif, Taimoor Afzal, Mohsin Abbas Khan, Muhammad Younus, Umair Khurshid, Maryem Safdar, Sohaib Saifulah, Bashir Ahmad, Abubakar Sufyan, Siddique Akber Ansari, Hamad M. Alkahtani, Irfan Aamer Ansari

**Affiliations:** ^1^ Faculty of Medicine and Allied Health Sciences, The Islamia University of Bahawalpur, Bahawalpur, Pakistan; ^2^ Rural Health Center, Punjab, Pakistan; ^3^ Department of Pharmaceutical Chemistry, Faculty of Pharmacy, The Islamia University of Bahawalpur, Bahawalpur, Pakistan; ^4^ Department of Pharmacognosy, Faculty of Pharmacy, The Islamia University of Bahawalpur, Bahawalpur, Pakistan; ^5^ Department of Livestock and Poultry Production, Bahauddin Zakariya University, Multan, Pakistan; ^6^ Department of Pharmaceutical Chemistry, College of Pharmacy, King Saud University, Riyadh, Saudi Arabia; ^7^ Department of Drug Science and Technology, University of Turin, Turin, Italy

**Keywords:** silver nanoparticles (AgNPs), *Piper cubeba*, enzyme inhibitory activity, anti-urease activity, spectroscopy, FTIR analysis, XRD, SEM

## Abstract

**Introduction:** The area of “Green Synthesis of Nano-medicine,” as compared to its synthetic counterparts, is a relatively safer research technology for various biomedical applications, including identification, therapeutic application, and prevention of pathological conditions, pain control, safety, and development of human wellness. The present study explored the synthesis and characterization of AgNPs using the ethanolic extract of *Piper cubeba* fruit as a reducing and stabilizing agent and its potential as an enzyme inhibitory agent. Urease inhibitors are helpful against many severe diseases, including gastric ulcers induced by Helicobacter pylori.

**Method:** The fruits of the *Piper cubeba* plant were taken and ground to a fine powder. Plant material was added to 500 ml ethanol, and the mixture was filtered. The solvent of the filtrate was evaporated, and a thick, gummy extract was obtained and stored at 4°C in the refrigerator. AgNPs were green synthesized from solutions of AgNO3 using the *P. cubeba* extract, which was indicated by a change in the color from light brown to deep brown. The synthesized AgNPs were characterized via Ultraviolet-visible (UV-Vis) spectroscopy, Fourier transform infrared (FTIR) spectroscopy, X-ray diffraction (XRD), and scanning electron microscopy (SEM).

**Results and Discussion:** Analysis showed the reduction of Ag+ to Ag0 at room temperature (25°C), and the average particle size of AgNPs was in the range of 40–80 nm. Consequently, the synthesized AgNPs were evaluated for their anti-urease activity. The maximum urease inhibition of the *Piper cubeba* ethanolic extract was 88.5% at 5 mg conc., and of derived nanoparticles was 78.6% at 0.05 mg conc. The results were nearly similar to the control drug, i.e., thiourea (0.5 and 0.6 mM conc., respectively).

**Conclusion:** The study concluded that the *P. cubeba* extract, as well as its green-derived AgNPs, might prove to be a better and safer substitute for their enzyme inhibitory potential in emerging medicine and novel drug delivery techniques to improve and maintain human health.

## 1 Introduction

In recent years, green production of metallic nanoparticles has emerged as a fresh and exciting area of study. The creation of green nanoparticles has grown significantly over the past several years due to its many advantages, including ease of scaling up for large-scale synthesis, low cost, high stability of the nanoparticles produced, and non-toxic byproducts (Liaqat et al., 2022). In comparison with conventional chemical or physical processes, green synthesis provides several benefits, such as the need for inoffensive reaction conditions, the use of fewer toxic components, affordability, and environmental friendliness. There is no need for high pressure, energy, temperature, or toxic chemicals in the green synthesis method, which makes it an economic tool for the production of beneficial nanoparticles (Malhotra and Alghuthaymi, 2022). Green synthesis has drawn attention to the production of different metal and metal oxide nanoparticles since chemical synthesis techniques result in the presence of harmful chemical species adsorbed on the surface of nanoparticles. Therefore, green synthesis approaches are found to be a more reliable and economic route to synthesize these metal nanoparticles (Kanagamani et al., 2019).

Nanotechnology is associated with the process that occurs on the nanometer scale, which is between 1 and 100 nm (Mulfinger et al., 2007). Nanoparticles have several applications in therapeutical, industrial, and biological fields. High pressure, energy, temperature, and toxic substances are required for the chemical and physical creation of nanoparticles. Plants and plant component extract-based biosynthesis has been demonstrated to be both cost-effective and environmentally benign in this aspect (Casida and Quistad, 2000).

Researchers are adopting green technologies for the synthesis of various metal nanoparticles in response to the growing need for environmentally friendly nanoparticles. Plant extracts, on the other hand, are now used as reducing and capping agents in the synthesis of nanoparticles, which may be preferable to photochemical reduction, heat evaporation, electrochemical reduction, and chemical reduction procedures (Veerasamy et al., 2011).

Plants are a valuable source of medicinal substances. Plant-derived medicines have appeared in medical fields as a result of folklore or traditional medicine’s use of plant materials as an indigenous remedy. Plant-derived medicine is currently used as the first line of basic healthcare for 80 percent of the world’s population (Mukeshwar et al., 2011). There are now several regulatory regimes for herbal medicines, including prescription medications, over-the-counter medicinal products, traditional remedies, and dietary supplements. It is necessary to harmonize and enhance regulatory procedures by combining scientific studies and traditional wisdom (Kamboj, 2000). The biological catalyst enzymes (also known as biologically active proteins) help living organisms speed up metabolic processes. It can also be isolated from cells and utilized to catalyze a wide range of essential industrial products (Robinson, 2015).

Enzymes catalyze nearly every cellular activity. Activators (positive modifiers) are chemicals that enhance enzyme activity, while inhibitors are substances that reduce enzyme activity (negative modifiers). Enzyme inhibitors are compounds that convert enzymes into inactive substances, slowing down the pace of an enzyme-catalyzed process. Urease (urea amidohydrolase) may be the first crystalline enzyme. It has also been demonstrated that urease may be a virulence factor important in a variety of disorders, including long-term infections (Sirko and Brodzik, 2000). Medically, urease is a significant virulence factor linked to the pathogenesis of various chemical conditions, for example, peptic ulceration and the development of infection-induced urinary stones. Urease inhibitors disintegrate crystals in the urine and prevent the production of new crystals (Watson, 2000).

Urease converts urea to ammonia and carbon dioxide; urease is an essential survival element for the human gastric mucosa in its acidic environment. Urea is a byproduct produced during normal kidney function in humans. The capacity of *H. pylori* urease to produce ammonia is approximately 100 times larger than *Proteus* organisms (Sirko and Brodzik, 2000). Enzyme inhibitors are specific chemical compounds with a low molecular weight. They can lower or entirely inhibit the catalytic ability of enzymes, either temporarily or entirely permanently. The enzyme inhibitors can occupy enzymes and obstruct their activity toward their actual substrates. Urease blockers can play an imperative role in contradicting the harmful aspect of urease in living life. Urease blockers are useful against numerous serious infections caused by *H. pylori’s* urease secretion, including gastric tract syndromes and urinary tract struvite urolithiasis (Amtul et al., 2004).

*P. cubeba* is a Piperaceae plant, which is extensively dispersed across the world, particularly in tropical and subtropical areas, and is a rich source of bioactive lignans and neolignans (Parmar et al., 1997). *P. cubeba* is characterized as a urinary system cleanser, and it is utilized for gonorrhea, genitourinary tract diseases and inflammation of urinary channels, and chronic bronchial catarrh because of its diuretic and stimulating qualities (Akbar, 2020).

The study’s goals are as follows: to develop and create a safe, eco-friendly, and inexpensive technique for the synthesis of Ag nanoparticles; characterize and synthesize silver nanoparticles with analytical methods; and estimate the enzyme inhibitory activity of silver nanoparticles of *P. cubeba.*

## 2 Materials and method

### 2.1 Chemicals and reagents

The reagents used in this research are of high-purity analytical grade. Reagents include silver nitrate (Merck; 99% pure), de-ionized water (obtained from the Phytomedicine Research Lab, University College of Conventional Medicine), double distilled water (Department of Pharmacy, The Islamia University of Bahawalpur), and ethanol (Merck; 99.9% pure). Glassware like beakers, conical flasks, measuring cylinders, burette, and pipette used was made up of Pyrex brand. A mercury thermometer (360°C) was also used to measure temperature.

Jack bean urease bought from Sigma-Aldrich (Sigma Co., St. Louis, United States) was used for the enzyme assay. Thiourea, alkali (0.1% w/v NaOCl and 0.5% w/v NaOH), and reagents (0.005% w/v sodium nitroprusside and 1% w/v phenol) were used as reagents and chemicals.

### 2.2 Plant collection and identification

The *Piper cubeba* plant was recognized by the Department of Botany, IUB, and the voucher’s identification number is “Ref. No. 42/Botany.” *P. cubeba* fruit was obtained at a local market in Bahawalpur. To remove dust and other impurities, the *P. cubeba* fruit sample was properly rinsed and washed twice with fresh water, followed by de-ionized water. The fruit was ground into fine powder and preserved in an air-tight, moisture-proof, and light-proof container at 25°C (Pandey and Savita, 2017) for further research.

### 2.3 Extraction of the plant

An amount of 100 g of the plant powder was steeped in 500 mL of pure ethanol. The ethanolic powder mixture was stirred for 3 h on a magnetic stirrer. After 48 h of immersion, the ground plant material was strained using a gauze fabric. To obtain a semitransparent filtrate, the filtrate was re-filtered with Whatman filter paper no 1. The powder residue was resoaked in 500 mL pure ethanol and swirled on a magnetic stirrer for 2–3 h. After 24 h of immersion, the resultant ethanolic combination was again filtered through a muslin cloth, and the obtained filtrate was filtered for the second time with Whatman filter paper no. 1. This procedure was then repeated three times in total. On the rotary evaporator, the solvent (ethanol) was evaporated by keeping the temperature of the water bath at around 35°C–40°C, and a vacuum pressure of 70 mbar combined with a condensation temperature around 4°C was applied to obtain the concentrated crude extract. The semi-solid mass of crude pharmaceuticals was produced after air drying the concentrated crude extract. This raw extract was kept in the refrigerator at 4°C for future use in the production of nanoparticles of diverse sizes.

### 2.4 Green synthesis

The *P. cubeba* extract was used as a reducing agent in the green synthesis of AgNPs of varied sizes from AgNO_3_, and it also aided to stabilize the formed AgNPs *via* its capping activity. By dissolving 0.0339 g of silver nitrate (AgNO_3_) in 20 mL of double distilled water, 0.01 M aqueous silver nitrate (AgNO_3_) solution was prepared. A measure of 20 mL of the 0.01 M AgNO_3_ solution was added to each conical flask, designated A–D, on a magnetic mixer. Then, using a burette, varying concentrations, i.e., 20, 30, 40, and 50 mg of the *P. cubeba* extract diluted in 20 mL of water, were added drop-by-drop to a corresponding flask with continual stirring to generate AgNPs of different sizes.

The sample colors changed from light brown to deep brown after 3 h of stirring at 25°C (room temperature), which demonstrated the synthesis of AgNPs physically. In addition, during the duration of the experiment, the samples were maintained in full darkness.

To check the conversion of Ag^+^ ions to Ag^0^, a UV spectrophotometer was used to authenticate the results. Following that, samples were centrifuged at 6,000 RPM for 10 min, and the pallet containing silver nanoparticles was washed with ethanol and sonicated. In the end, by evaporating the ethanol at room temperature, the powdered AgNP samples were completely dried, weighed, and stored at 4°C.

### 2.5 Characterizations of AgNPs

AgNPs were characterized by reliable techniques such as Ultraviolet–visible spectroscopy (UV), X-Ray diffraction (XRD), Fourier transform infrared (FTIR) spectroscopy, and scanning electron microscopy (SEM).

#### 2.5.1 Ultraviolet–visible spectroscopy (UV-vis)

The reduction of silver ions and the synthesis of AgNPs were evaluated by measuring the UV-visible absorbance of the reaction mixture without dilution by ultraviolet-visible spectroscopy. By monitoring a peak, spectrometry is used to validate the synthesis of AgNPs.

#### 2.5.2 Fourier transform infrared (FTIR) spectroscopy

FTIR is a procedure for acquiring high-resolution FTIR spectra of liquid or solid materials. It indicates the presence of functional phytochemical groupings in the samples. The reducing and stabilizing functional groups are characterized using this method.

#### 2.5.3 X-ray diffraction (XRD)

XRD spectroscopy was utilized to detect the size and crystal structure of nanoparticles. The Debye–Scherrer equation was used to compute it.

L=Kλ/βcos⁡θ.

In this scenario,

K = Scherrer constant

*λ* = X-ray wavelength

*θ* = half-width of the peak and *β* = half of the Bragg angles. With the use of this equation, the size (L) of nanoparticles could be simply estimated (Li et al., 2007).

#### 2.5.4 Scanning electron microscopy (SEM)

After the green synthesis of AgNPs, samples were then subjected to SEM examination in order to examine their morphology. This method also supplied information on the size and shape of the AgNPs. The sample was prepared by placing a drop of silver nanoparticles on a carbon-coated copper grid and subsequently air-dried before transferring it to the microscope (Devi et al., 2013). Since the silver itself is conductive in nature and the produced AgNPs were stable, the sample did not require any additional gold/carbon coating.

#### 2.5.5 Urease inhibitory assay of the extract and AgNPs

The urease inhibitory experiment was performed on the extract fractions of *P. cubeba* and their respective NPs of *P. cubeba*. With minimal changes, the previously stated technique was used for this investigation (Weatherburn, 1967). The experiment was performed in triplicates (*n* = 3) for each sample. The fractions of the crude extract and their associated NPs were separately added to 96-well plates and incubated for 30 min at 30°C using 5 µL of standard solutions (0.5–0.00625 mM concentrations). The experiment materials (NPs and fractions) were put into reaction mixes that included 55 µL buffer (pH 6.8), jack bean urease (25 µL), and 100 mM urea. Various concentrations of the samples, 0.5 mM (control), 0.625, 1.25, 2.5, and 5 mg of *P. cubeba* crude extract, and 0.6 mM (control) and 0.05 mg of *P. cubeba* AgNPs were employed to investigate the kinetics. For that purpose, each well received 70 µL of alkali (0.1% w/v NaOCl and 0.5% w/v NaOH) and 45 µL of phenol reagents (0.005% w/v sodium nitroprusside and 1% w/v phenol). After 1 h, the absorbance was measured at 630 nm. Using the indophenol method and thiourea as a standard, the production of ammonia (NH_3_) was investigated. Finally, MS-Excel, SoftMax Pro (Molecular Devices, CA, United States), and EZ-FIT software applications were used to analyze the data. The following formula was used to calculate the percentage urease inhibition of each sample (Weatherburn, 1967):

% Inhibition=100−O.D.test/O.D.control_×100.

#### 2.5.6 Determination of IC_50_ values

IC_50_ values (half-maximal inhibitory concentrations) were established by comparing the inhibitory activity of the *Piper cubeba* extract to a positive control using spectrophotometric analysis. The IC_50_ values were calculated using linear regression curves.

## 3 Results and discussion

### 3.1 Characterization of AgNPs

Physical proof for the production of AgNPs is the change in color of the AgNO_3_ solution from white to dark brown after adding the *P. cubeba* ethanolic extract (Banerjee et al., 2014; Balachandar et al., 2019). The color shift was induced by the stimulation of surface plasmon resonance (excitation), which specifies the creation of AgNPs by reducing Ag^+^ to Ag^0^ (Saxena et al., 2012; Reddy et al., 2021).

#### 3.1.1 UV-visible spectroscopy

**FIGURE 1:**
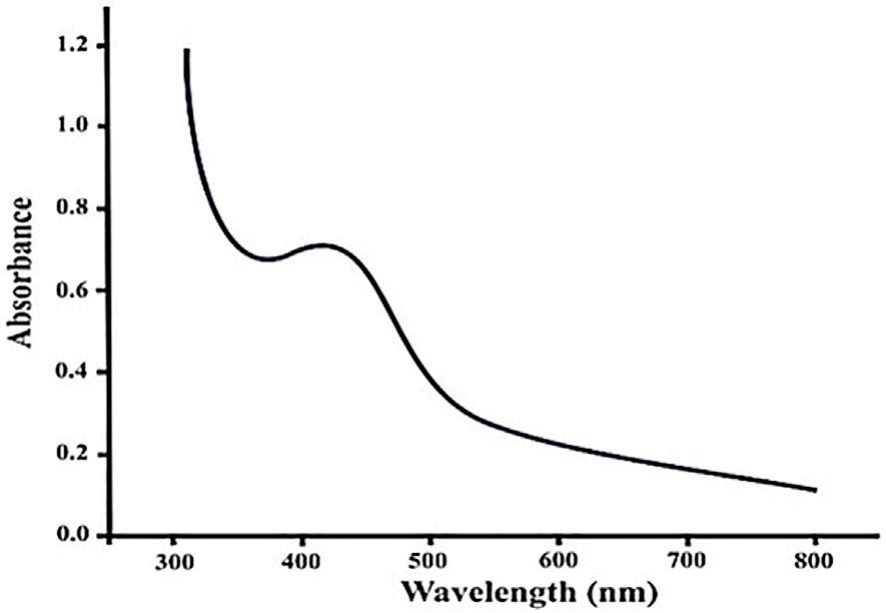
UV-visible spectra of silver nanoparticles of *Piper cubeba*.

UV-visible spectra were obtained for the AgNP sample. Deionized water was used as a control for each sample. Peaks in the region of 423–430 nm for the samples are shown in the Figure 1. According to the reports, silver nanoparticles are frequently discovered to show a plasmon resonance absorption peak because of surface plasmon vibration excitation in the visible region of 380–500 nm. The UV-vis spectroscopy experiment was performed to ensure that the AgNPs generated were stable. Data were collected every 24 h for a week. The UV-vis spectrum is seen in the diagram.

Nanoparticle concentration, size, type, and shape all impact the surface plasmon resonance. Under visible light, silver NPs exhibit a plasmon resonance absorption peak. Surface plasmon waves excite at the wavelength range of 380–440 nm (Chouhan and Meena, 2015; Ahmad et al., 2022).

#### 3.1.2 FTIR analysis

**FIGURE 2:**
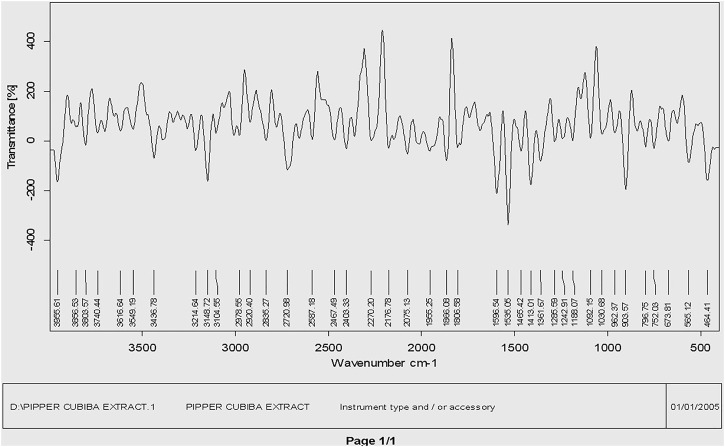
FTIR spectra of the *Piper cubeba* crude extract.

**FIGURE 3:**
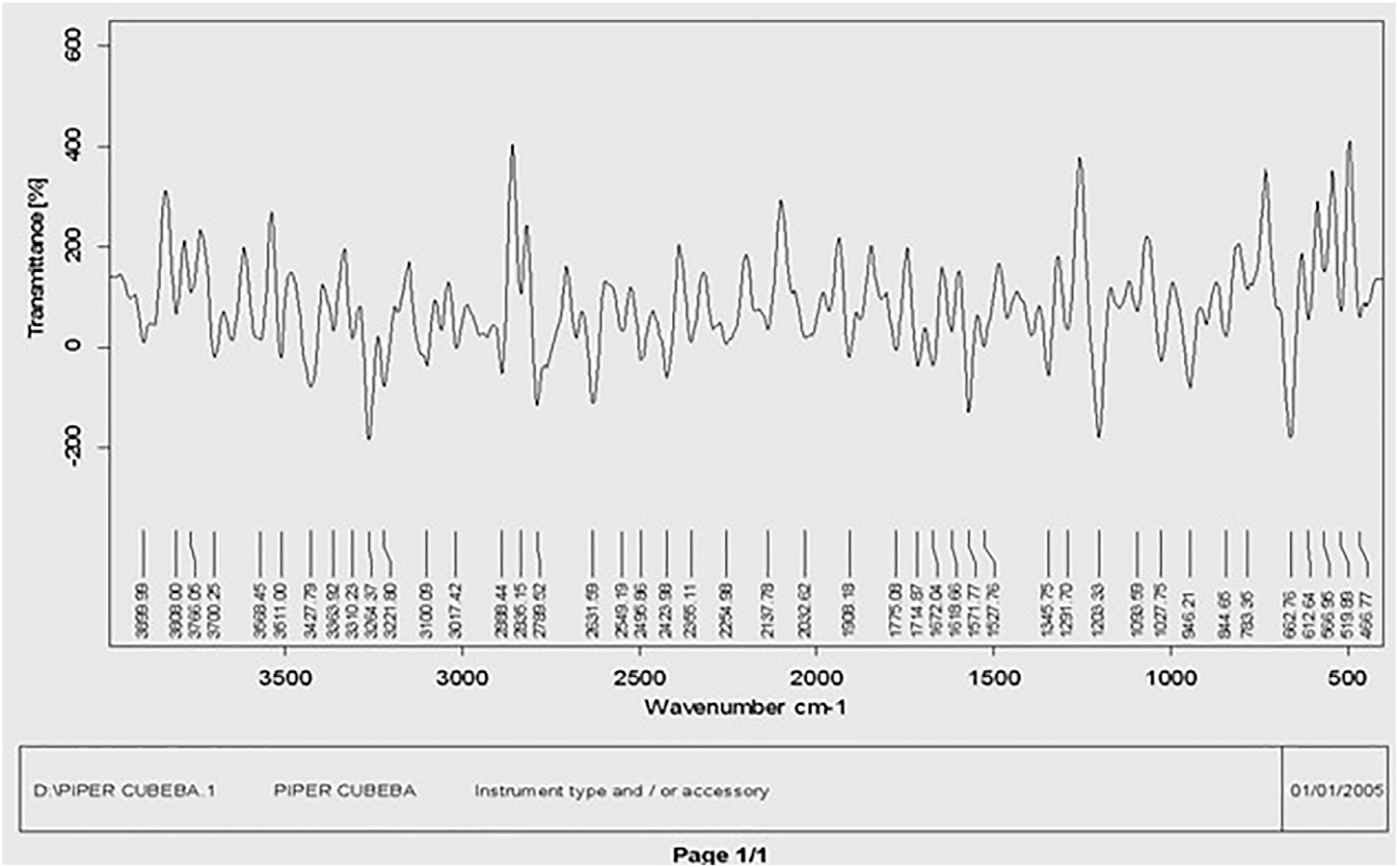
FTIR spectra of silver nanoparticles synthesized from the *Piper cubeba* extract.

The main phytochemical components and bioactive chemicals in the *P. cubeba* ethanol extract were identified using an FTIR spectrum (Figure 2, 3). The chemicals detected in the extract are oxidized by silver ions, reducing the Ag^+^ ions to Ag^0^ and stabilizing the generated silver NPs. The outcomes of FTIR analysis of this study give different stretches of the bond at altered peaks; 3770.91, 3699.19, and 3163.40 cm^−1^ (O-H stretch); C-O stretching of primary alcohol at 1066 cm^−1^; 2753.96 cm^−1^ stretch for −CH=O (single aldehyde); 2362.09 cm^−1^ for stretching; *α, β*-unsaturated ketone (1603.67 cm^−1^); 1539.82 cm^−1^ (C-O-O stretch) and 1559.13 cm^−1^ (C-H stretch), peak at 1470.47 cm^−1^ for alkane gem dimethyl, 1387.72 cm^−1^ C-H bending alkane stretch, 1260.42 cm^−1^ alkyl aryl ether (C-O-C) stretch, and 1212.65 cm^−1^ vinyl ether (H2C=HC-O) stretch. The figure shows the medium and sharp peaks near 3699.19 and 3770.91 cm^−1^, which indicate the presence of free alcoholic groups, while the peak at 3163.40 cm^−1^ determines the presence of the bonded –OH group in compounds of the extract. The peak near 829 cm^−1^ corresponds to C=CH_2_, and the peaks at 1603.67 cm^−1^ assign to (–C=C-) *α, β*-unsaturated ketones (Devaraj et al., 2013; Mustapha et al., 2022). The presence of the aforementioned functional groups, as well as the presence of the alcohol groups, is obvious in the compounds discovered from *P. cubeba*; unsaturated groups and alkyl aryl ethers are examples of functional groups.

#### 3.1.3 SEM analysis

**FIGURE 4:**
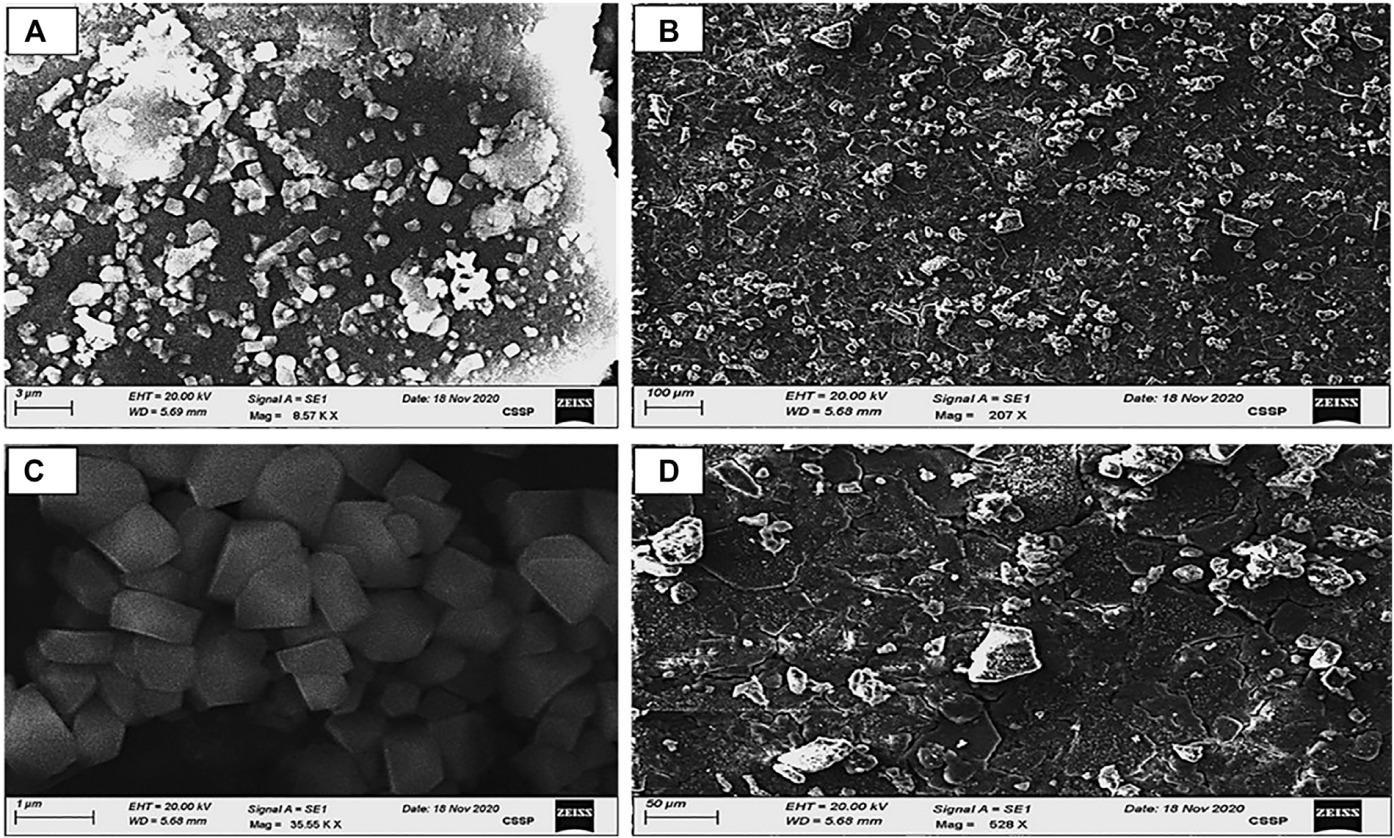
SEM images of AgNP samples **(A–D)**; calculated grain size area: **(A)** = 40 nm, **(B)** = 50 nm, **(C)** = 65 nm, and **(D)** = 79 nm obtained by 0.01 AgNO_3_ derived from varied concentrations of the *Piper cubeba* extract at room temperature.

The morphology of these biosynthesized silver NPs was studied by SEM. It is also used to determine the particle size. The figure depicts SEM figures of the generated AgNPs, in addition to their sizes and structures. Silver nanoparticles can be an irregular square shape. Clusters have appeared in large numbers. AgNPs were found to be between 40 and 80 nm in size on average. As the concentration of the plant extract increases, the size of the nanoparticle grows, as illustrated in the figures. The particles were seen in SEM imaging (Figure 4).

Smaller silver nanoparticles have accumulated, resulting in bigger silver nanoparticles in SEM (Awwad and Salem, 2012). The researchers discovered that increasing the extract concentration reduced the number of silver ions while increasing the particle size. This trend has been seen by other studies as well (Song and Kim, 2009).

#### 3.1.4 XRD analysis

**FIGURE 5:**
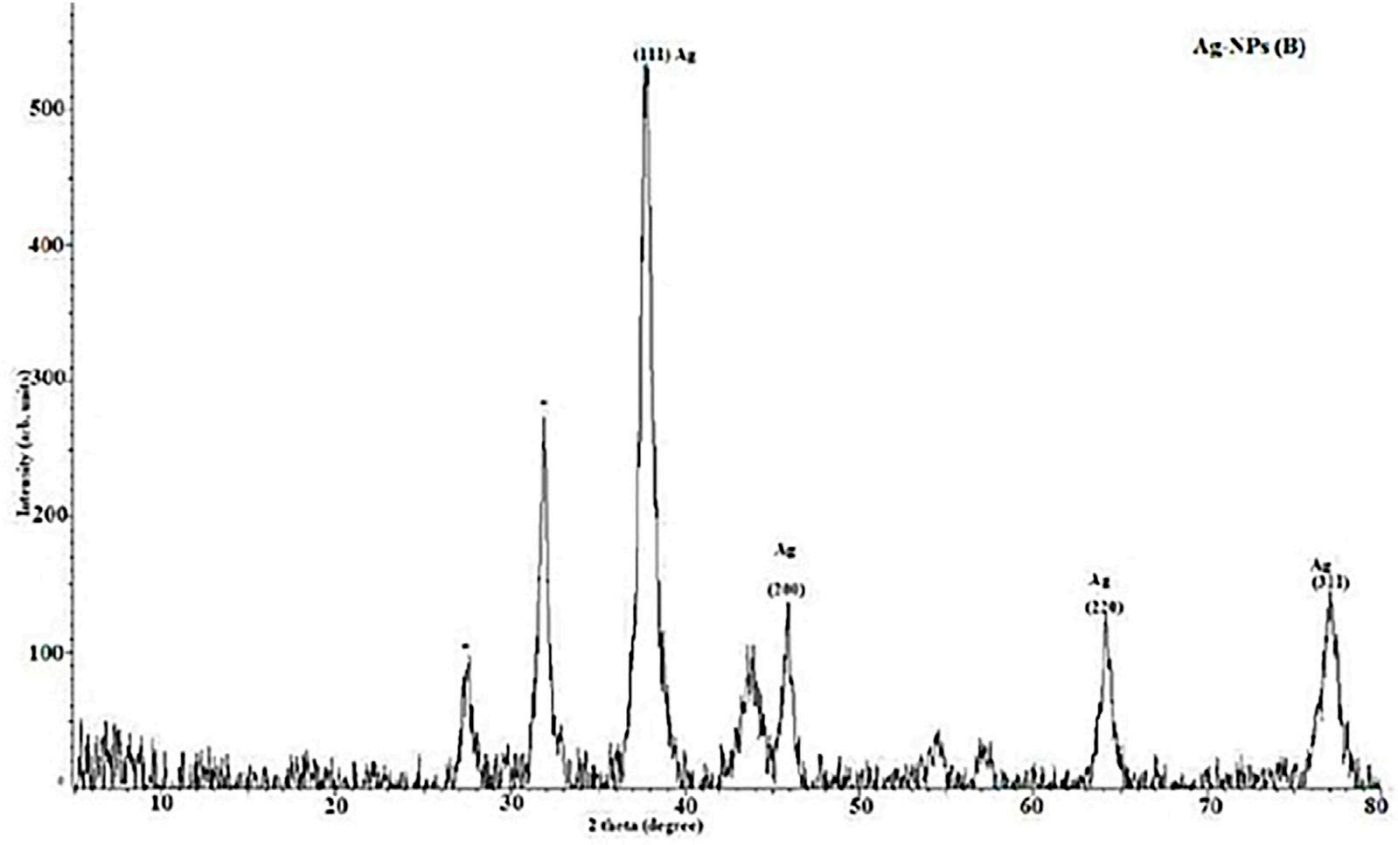
XRD of silver nanoparticles synthesized from the *Piper cubeba* extract.

The XRD patterns of synthetic AgNPs generated using the *P. cubeba* extract are shown in the Figure 5. Four significant diffraction spikes arise in 111, 200, 220, and 311 planes at 37.6°, 45.8°, 64.18°, and 77.1°, respectively. The face-centered cubic formation of synthesized AgNPs of various dimensions may be seen in these planes. The unassigned peak at 31.9° was considered to have formed because of the crystallization of other organic compounds in the *P. cubeba* extract. The results confirmed the crystalline characteristic and face-centered-cubic structure of the produced AgNPs. These peaks correspond to the synthesized AgNP reference card (JCPDS Card No. 4-0783) (Bharathi et al., 2018). The Debye–Scherrer equation was applied to compute the crystalline nature of the particles.

The mean crystalline diameters of AgNPs were determined to be 7, 15, 38, and 51 nm. The crystalline nature of AgNPs was reflected by these XRD peaks.

#### 3.1.5 Urease inhibitory assay of the extract and AgNPs

Results showed 55.3%, 68.7%, 79.2%, and 88.5% urease inhibition at various concentrations of the *P. cubeba* crude extract, i.e., 0.625, 1.25, 2.5, and 5 mg, and 95.2% inhibition of positive control (thiourea) at 0.5 mM concentration. Similarly, *P. cubeba* AgNPs showed 78.6% urease inhibition activity at 0.05 mg concentration which was compared to the percent urease inhibition of the positive control (thiourea), which was 90.2% at 0.6 mM concentration.

##### 3.1.5.1 Determination of IC_50_ values

**FIGURE 6:**
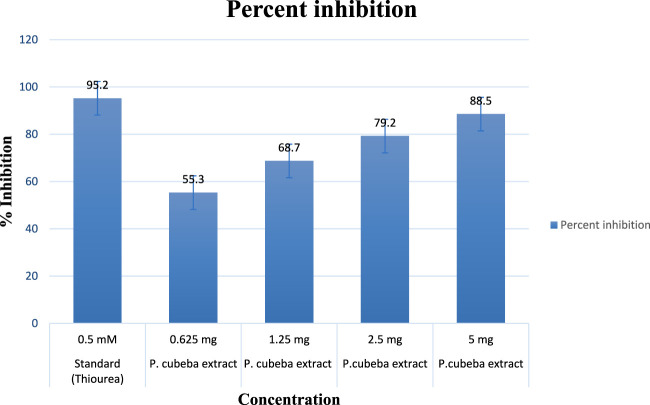
Percentage inhibition of the *P. cubeba* extract.

**FIGURE 7:**
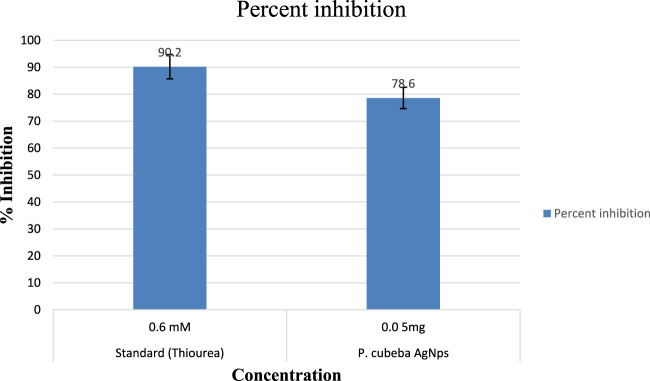
Percentage inhibition of *P. cubeba*-derived AgNPs.

Results showed IC_50_ values (half-maximal inhibitory concentrations) 20 mg/ml ± 0.12 of the standard (thiourea), 2.5 mg/ml ±0.5,0.65 mg/ml ± 0.04, 0.35 mg/ml ± 0.01, and 0.15 mg/ml ± 0.001 of various concentrations of the *P. cubeba* crude extract that were established by comparing the inhibitory activities, i.e., 95.2% (control), 55.3%, 68.7%, 79.2%, and 88.5% of respective *P. cubeba* extract fractions 0.625, 1.25, 2.5 and 5 mg and positive control (0.5 mM) using spectrophotometric analysis. The IC_50_ values were calculated using linear regression curves, as shown in the graph (Figure 6). Similarly, IC_50_ values of silver NPs and positive control (thiourea) were 9.3 mg/ml ± 0.05 and 0.002 mg/ml ± 0.001, respectively, which was established by comparing the inhibitory activities 90.2% and 78.6% of positive control (0.6 mM) and *P. cubeba* AgNPs (0.05 mg), respectively (Figure 7).

## 4 Conclusion

In this work, AgNPs were effectively prepared utilizing an ethanolic extract of *P. cubeba*, with UV-Vis, XRD, and SEM analyses explaining the reduction of Ag^+^ to Ag^0^ at room temperature. According to FTIR research, the *P. cubeba* extract comprises chemicals that aid the reduction of silver into NPs in addition to their ability to stabilize these particles. The green AgNP synthesis process is a cost-effective and environmentally friendly technology. The synthesized AgNPs were round and spherical in shape, with diameters varying from 40 to 79 nm, according to SEM analysis.

The urease inhibitory action of *P. cubeba* was equivalent to that of thiourea, which was used as a control. Standard thiourea inhibited urease by 95.2%, with an IC_50_ of 20 ± 0.12. The *P. cubeba* extract has the highest urease inhibitory action, i.e., 88.5% (IC_50_: 0.15 mg/ml ± 0.001). The IC_50_ values of the standard (thiourea) and the extracts (*P. cubeba*) were calculated by graphing the percentage inhibition than the concentration of the standard and extracts, respectively.

The urease inhibitory action of *P. cubeba* was equivalent to that of thiourea, which was used as a control. Standard thiourea inhibited urease by 90.2%, with an IC_50_ value of 9.3 ± 0.05. The *P. cubeba* silver nanoparticles have the highest urease inhibitory action, i.e., 78.6% (IC_50_: 0.002 mg/ml ± 0.001). The IC_50_ values of the standard (thiourea) and AgNPs (*P. cubeba*) were calculated by graphing the percentage inhibition than the concentration of the standard and extracts, respectively.

As a result, the study concluded that *P. cubeba* may, perhaps, be utilized as an enzyme inhibitor in medicine to improve or maintain human health. The findings were also scientifically evaluated, confirming the use of traditional medicinal herbs for the management and treatment of ulcer and UTI infections. Therefore, based on the prior explanation, we can conclude that *P. cubeba* can be utilized as an alternative medication for illness prevention and therapy.

## Data Availability

The original contributions presented in the study are included in the article/Supplementary Material; further inquiries can be directed to the corresponding author.

## References

[B1] Ahmad N. Jabeen M. Haq Z. U. Ahmad I. Wahab A. Islam Z. U. (2022). Green fabrication of silver nanoparticles using Euphorbia serpens Kunth aqueous extract, their characterization, and investigation of its *in vitro* antioxidative, antimicrobial, insecticidal, and cytotoxic activities. BioMed Res. Int. 2022, 1–11. 10.1155/2022/5562849 PMC8763500 35047637

[B2] Akbar S. (2020). Handbook of 200 medicinal plants: A comprehensive review of their traditional medical uses and scientific justifications.

[B3] Amtul Z. Rasheed M. Choudhary M. I. Rosanna S. Khan K. M. Atta-ur-Rahman (2004). Kinetics of novel competitive inhibitors of urease enzymes by a focused library of oxadiazoles/thiadiazoles and triazoles. Biochem. biophysical Res. Commun. 319, 1053–1063. 10.1016/j.bbrc.2004.05.036 15184088

[B4] Awwad A. M. Salem N. M. (2012). Green synthesis of silver nanoparticles byMulberry LeavesExtract. Nanosci. Nanotechnol. 2, 125–128. 10.5923/j.nn.20120204.06

[B5] Balachandar R. Gurumoorthy P. Karmegam N. Barabadi H. Subbaiya R. Anand K. (2019). Plant-mediated synthesis, characterization and bactericidal potential of emerging silver nanoparticles using stem extract of Phyllanthus pinnatus: A recent advance in phytonanotechnology. J. Clust. Sci. 30, 1481–1488. 10.1007/s10876-019-01591-y

[B6] Banerjee P. Satapathy M. Mukhopahayay A. Das P. (2014). Leaf extract mediated green synthesis of silver nanoparticles from widely available Indian plants: Synthesis, characterization, antimicrobial property and toxicity analysis. Bioresour. Bioprocess. 1, 3–10. 10.1186/s40643-014-0003-y

[B7] Bharathi D. Diviya Josebin M. Vasantharaj S. Bhuvaneshwari V. (2018). Biosynthesis of silver nanoparticles using stem bark extracts of Diospyros Montana and their antioxidant and antibacterial activities. J. Nanostructure Chem. 8, 83–92. 10.1007/s40097-018-0256-7

[B8] Casida J. E. Quistad G. B. (2000). Insecticide targets: Learning to keep up with resistance and changing concepts of safety. J. Appl. Biol. Chem. 43, 185–191.

[B9] Chouhan N. Meena R. (2015). Biosynthesis of silver nanoparticles using Trachyspermum ammi and evaluation of their antibacterial activities. Int. J. Pharma Biol. Sci. 62, 1077–1086.

[B10] Devaraj P. Kumari P. Aarti C. Renganathan A. (2013). Synthesis and characterization of silver nanoparticles using cannonball leaves and their cytotoxic activity against MCF-7 cell line. J. Nanotechnol. 2013, 1–5. 10.1155/2013/598328

[B11] Devi J. S. Bhimba B. V. Peter D. M. (2013). Production of biogenic silver nanoparticles using Sargassum longifolium and its applications. Indian J. Geo-Marine Sci. 42 (1), 125–130.

[B12] Kamboj V. P. (2000). Herbal medicine. Curr. Sci. 78, 35–39.

[B13] Kanagamani K. Muthukrishnan P. Shankar K. Kathiresan A. Barabadi H. Saravanan M. (2019). Antimicrobial, cytotoxicity and photocatalytic degradation of norfloxacin using Kleinia grandiflora mediated silver nanoparticles. J. Clust. Sci. 30, 1415–1424. 10.1007/s10876-019-01583-y

[B14] Li S. Shen Y. Xie A. Yu X. Qiu L. Zhang L. (2007). Green synthesis of silver nanoparticles using Capsicum annuum L. extract. Green Chem. 9, 852–858. 10.1039/b615357g

[B15] Liaqat N. Jahan N. Anwar T. Qureshi H. (2022). Green synthesized silver nanoparticles: Optimization, characterization, antimicrobial activity, and cytotoxicity study by hemolysis assay. Front. Chem. 10, 952006. 10.3389/fchem.2022.952006 36105303 PMC9465387

[B16] Malhotra S. P. K. Alghuthaymi M. A. (2022). Biomolecule-assisted biogenic synthesis of metallic nanoparticles. Agri-Waste Microbes Prod. Sustain. Nanomater. 1, 139–163. 10.1016/b978-0-12-823575-1.00011-1

[B17] Mukeshwar P. Mousumi D. Shobit G. Surender K. C. (2011). Phytomedicine: An ancient approach turning into future potential source of therapeutics. J. Pharmacogn. phytotherapy 3, 27–37.

[B18] Mulfinger L. Solomon S. D. Bahadory M. Jeyarajasingam A. V. Rutkowsky S. A. Boritz C. (2007). Synthesis and study of silver nanoparticles. J. Chem. Educ. 84, 322. 10.1021/ed084p322

[B19] Mustapha T. Misni N. Ithnin N. R. Daskum A. M. Unyah N. Z. (2022). A review on plants and microorganisms mediated synthesis of silver nanoparticles, role of plants metabolites and applications. Int. J. Environ. Res. Public Health 19, 674. 10.3390/ijerph19020674 35055505 PMC8775445

[B20] Pandey A. Savita R. (2017). Harvesting and post-harvest processing of medicinal plants: Problems and prospects. Pharma Innovation J. 6, 229–235.

[B21] Parmar V. S. Jain S. C. Bisht K. S. Jain R. Taneja P. Jha A. (1997). Phytochemistry of the genus piper. Phytochemistry 46, 597–673. 10.1016/s0031-9422(97)00328-2

[B22] Reddy N. Li H. Hou T. Bethu M. Ren Z. Zhang Z. (2021). Phytosynthesis of silver nanoparticles using perilla frutescens leaf extract: Characterization and evaluation of antibacterial, antioxidant, and anticancer activities. Int. J. nanomedicine 16, 15–29. 10.2147/ijn.s265003 33447027 PMC7802595

[B23] Robinson P. K. (2015). Enzymes: Principles and biotechnological applications. Essays Biochem. 59, 1–41. 10.1042/bse0590001 26504249 PMC4692135

[B24] Saxena A. Tripathi R. Zafar F. Singh P. (2012). Green synthesis of silver nanoparticles using aqueous solution of Ficus benghalensis leaf extract and characterization of their antibacterial activity. Mater. Lett. 67, 91–94. 10.1016/j.matlet.2011.09.038

[B25] Sirko A. Brodzik R. (2000). Plant ureases: Roles and regulation. Acta Biochim. Pol. 47, 1189–1195. 10.18388/abp.2000_3972 11996109

[B26] Song J. Y. Kim B. S. (2009). Rapid biological synthesis of silver nanoparticles using plant leaf extracts. Bioprocess Biosyst. Eng. 32, 79–84. 10.1007/s00449-008-0224-6 18438688

[B27] Veerasamy R. Xin T. Z. Gunasagaran S. Xiang T. F. W. Yang E. F. C. Jeyakumar N. (2011). Biosynthesis of silver nanoparticles using mangosteen leaf extract and evaluation of their antimicrobial activities. J. saudi Chem. Soc. 15, 113–120. 10.1016/j.jscs.2010.06.004

[B28] Watson C. J. (2000). “Urease activity and inhibition-principles and practice,” in Proceedings-international fertiliser society (York, United Kingdom: International Fertiliser Society), 1–40.

[B29] Weatherburn M. (1967). Phenol-hypochlorite reaction for determination of ammonia. Anal. Chem. 39, 971–974. 10.1021/ac60252a045

